# Supplementary material for: Discovery and pharmacophoric characterization of chemokine network inhibitors using phage-display, saturation mutagenesis and computational modelling
Source: Nat Commun. 2023 Sep 16;14:5763. doi: 10.1038/s41467-023-41488-z (PMC10505172; doi:10.1038/s41467-023-41488-z)
Supplement: Supplementary file 4 — Source Data [file 41467_2023_41488_MOESM4_ESM.zip › SourceData/scripts/xoctet_20230808081637.tar!/xoctet/analysis/crossbinding/SV_E7_E8_crossbinding_plot.pdf]

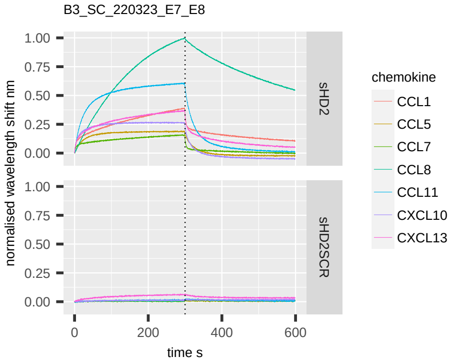
B3_SC_220323_E7_E8
normalised wavelength shift nm
1.00
0.75
0.50
0.25
0.00
sHD2
sHD2SCR
0
200
400
600
time s
chemokine
CCL1
CCL5
CCL7
CCL8
CCL11
CXCL10
CXCL13
